# Biotechnological Approach to Increase Oxyresveratrol Production in Mulberry In Vitro Plants under Elicitation

**DOI:** 10.3390/plants12030546

**Published:** 2023-01-25

**Authors:** Ana Belén Sabater-Jara, Lorena Almagro, Isabel Nicolás Sánchez, María Ángeles Pedreño

**Affiliations:** Department of Plant Biology, Faculty of Biology, University of Murcia, Campus de Espinardo, E-30100 Murcia, Spain

**Keywords:** cyclodextrins, in vitro plants, methyl jasmonate, *Morus alba*, oxyresveratrol, resveratrol

## Abstract

*Morus alba* L. is used for a range of therapeutic purposes in Asian traditional medicine, and its extracts are reported to be effective against lipidemia, diabetes, and obesity, as well as being hepatoprotective and tyrosinase-inhibitory. They are also included in cosmetic products as anti-aging and skin-whitening agents. Stilbenes, the major bioactive compounds found in *M. alba*, have received renewed attention recently because of their putative activity against COVID-19. In this study *M. alba* plants were established *in vitro*, and the effect of elicitation on plant growth and stilbene accumulation, specifically oxyresveratrol and *trans*-resveratrol, was investigated. Different concentrations of the elicitors including methyl jasmonate and cyclodextrins were applied, and stilbene levels were determined in leaves, roots, and the culture medium. Elicitation of the *M. alba* plants with 5 mM cyclodextrins, alone or in combination with 10 µM methyl jasmonate, significantly increased the total phenolic content in the culture medium and leaves after 7 days of treatment. The higher total phenolic content in the roots of control plants and those treated only with methyl jasmonate indicated that cyclodextrins promoted metabolite release to the culture medium. Notably, the cyclodextrin-treated plants with the highest levels of oxy- and *trans*-resveratrol also had the highest total phenolic content and antioxidant capacity. These results indicate that elicited *M. alba* in vitro plants constitute a promising alternative source of bioactive stilbenes to supply pharmaceutical and cosmeceutical industries.

## 1. Introduction

*Morus alba* is an economically important arboreal species of the family Moraceae. Widely cultivated in India, China, and other Asian countries, its leaves are used to feed silkworms and livestock [1,2]. Interest in *M. alba* has been increasing due to the wide-ranging health effects of its bioactive compounds. These are mainly secondary metabolites that protect the plant against environmental stress and are required for growth and development [3,4]. *M. alba* is particularly rich in phenolic compounds, which are found in leaves, stems and roots. Flavonoids are predominant (57.8%), followed by benzofurans (17.9%), phenolic acids (10.7%), coumarins (3.6%), chalcones (2.9%) and stilbenes (0.7%) [5].

**Figure 1:**
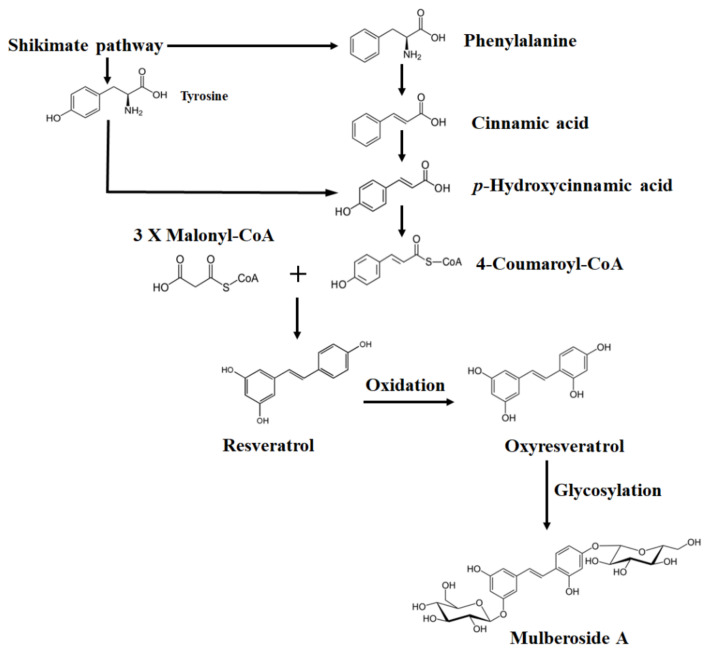
Biosynthetic pathway of resveratrol, oxyresveratrol, and mulberoside A.

Due to their antioxidant and anti-inflammatory activity, phenolic compounds have a wide variety of therapeutic effects including the prevention and treatment of cancer, obesity, diabetes, and liver, neurodegenerative, immunological, and cardiovascular diseases [6,7,8]. Despite containing a low proportion of stilbenes, mainly resveratrol and its derivative oxyresveratrol (Figure 1), *M. albus* has proved to be a promising source of these bioactive compounds for the health product industry. Pharmacological properties reported for stilbenes include anti-lipidemic [5], anti-diabetes [9], hepatoprotective [10], anti-obesity [11], and anti-tyrosinase effects [12]. In addition, due to their antioxidant activity, these metabolites are used as active ingredients in anti-aging and skin-whitening cosmetic products [13,14,15,16]. Moreover, stilbene-based natural compounds have recently been postulated as promising drug candidates against COVID-19 and other emerging respiratory viral infections [17,18].

The production and accumulation of these valuable secondary metabolites in white mulberry trees is limited by environmental, geographic, or seasonal factors [19]. In contrast, plant in vitro culture systems with controllable conditions can provide a reliable and continuous supply of target compounds, especially when combined with elicitation strategies. Stilbene biosynthesis can be enhanced by different biotic and abiotic elicitors, which induce the expression of key biosynthetic genes [20,21]. For example, stilbene production in *Vitis vinifera* L. has been increased by the application of signaling molecules such as methyl jasmonate (MJ) [22]. Elicitation with jasmonates activates defense mechanisms in plant cell cultures, involving a reprogramming of gene expression that induces stilbene production [23,24]. Our group has established a biotechnological method to increase the production of *trans*-resveratrol based on the elicitation of *Vitis* cell cultures with β-cyclodextrins (CDs) (Patent WO2010049563 A1; [25,26,27]). CDs also modified the expression profile of stilbene biosynthetic genes in *V. vinifera* cells [28,29]. Therefore, as well as sequestering and secreting metabolites, allowing them to be harvested from aqueous media without biomass destruction, CDs can activate their biosynthesis [30]. Additionally, CDs and MJ applied together exerted a synergistic effect on the biosynthesis of resveratrol and indole alkaloids in *V. vinifera* and *Catharanthus roseus* cell cultures, respectively, the enhanced production being highly correlated with an upregulation of biosynthetic genes [24,25].

Elicited *M. alba* in vitro cultures have also proved to be effective stilbene-producing systems [31,32,33], with the highest levels of mulberroside A, oxyresveratrol and resveratrol achieved in *M. alba* root cultures treated with MJ plus yeast extract [32]. The same treatment also improved the production of all three stilbenes in immobilized *M. alba* cells [33]. Oxyresveratrol and resveratrol production in *M. alba* callus cultures was increased by the application of 2-hydroxypropyl-β-CDs [34]. These results indicate that *M. alba* in vitro cultures can produce stilbenoids and have the potential to be developed into industrial-scale production systems.

In the light of the above, the aim of the present work was to study the effect of CDs and/or MJ on the accumulation of stilbenes in *M. alba* plants cultured *in vitro*. Additionally, the antioxidant capacity of extracts enriched in bioactive compounds obtained from the elicited *M. alba* cultures was evaluated.

## 2. Results and Discussion

### 2.1. Effect of Elicitors on Growth of M. alba In Vitro Plants

**Figure 2:**
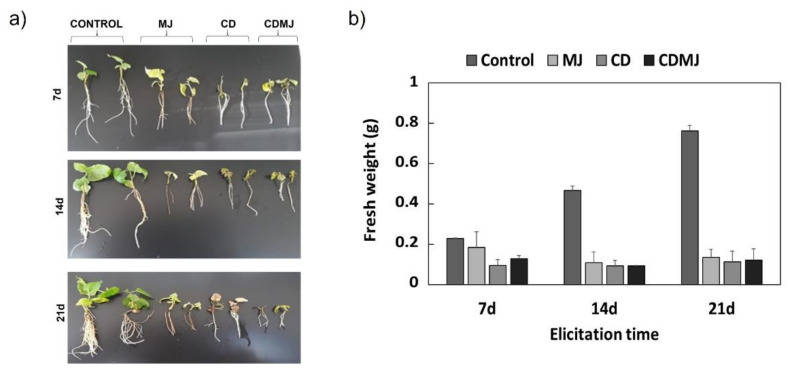
Effect of 50 mM cyclodextrins (CDs) and 100 µM methyl jasmonate (MJ), applied jointly or separately, on the growth (**a**) and biomass accumulation (**b**) of *Morus alba* in vitro plants at 7, 14 and 21 days of elicitation.

The growth pattern of *M. alba* in vitro plants in the presence of elicitors (50 mM CDs, 100 µM MJ, separately or in combination) was studied for 21 days (Figure 2). As shown in Figure 2, elicitation with CDs and/or MJ provoked a clearly phytotoxic effect in plant growth, with a significant reduction in leaf and root development with respect to the control at days 7, 14 and 21 of treatment.

In agreement with our results, Shabania et al. [35] reported that 2 mM MJ applied to *Glycyrrhiza glabra* in vitro cultures provoked a significant decrease in growth at 24 h, possibly because direct exposure to MJ at this concentration in the culture medium was toxic for root growth. At concentrations higher than 10 µM, MJ also reduced root or shoot growth in other species such as *Panax*, *Centella*, *Bupleurum* and *Bacopa monnieri* [36,37,38,39]. Similarly, MJ reprograms secondary metabolism and inhibits growth in plant cell cultures [24], which may be the result of metabolic competition between defense- and growth-related processes in response to elicitation [40,41].

### 2.2. Effect of Elicitors on the Total Phenolic Content in the Culture Medium of M. alba In Vitro Plants

**Figure 3:**
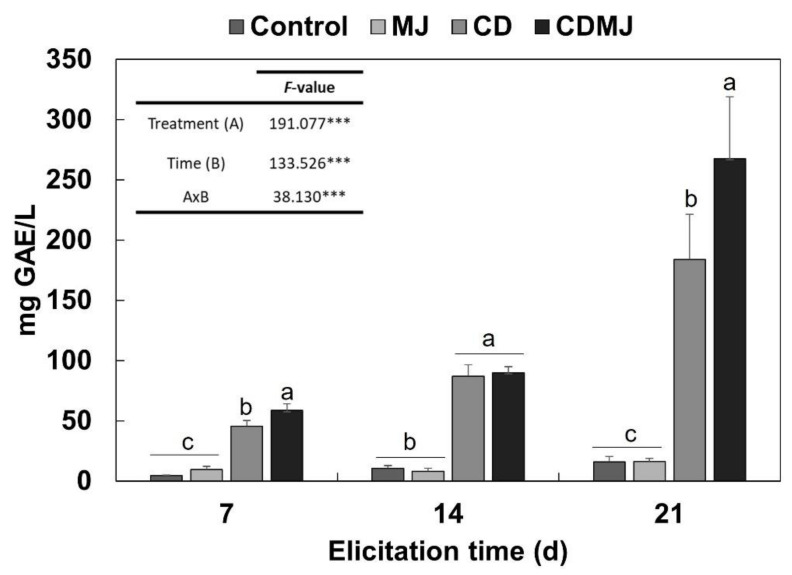
Effect of 50 mM cyclodextrins (CDs) and 100 µM methyl jasmonate (MJ), separately or in combination (CDMJ), on total extracellular phenolic compounds in *M. alba* in vitro plants after 7, 14 and 21 days of elicitation. Bars represent data expressed as mg gallic acid equivalent (GAE) /L. Letters denote statistically significant differences between the treatments at each elicitation time according to the Tukey test (*p* < 0.05). Figure shows F-values from two-way ANOVA significant at the 99.9% (***) level of probability.

The TPC of *M. alba* in vitro cultures was determined using the Folin–Ciocalteu method. Thus, after elicitation with 50 mM CDs and/or 100 µM MJ, the extraction and quantification of the TPC was carried out in the culture medium. ANOVA analysis showed that the TPC was strongly influenced by the treatment with CDs or CDMJ and time of elicitation, all differences being significant with a *p*-value < 0.001. As shown in Figure 3, the TPC of the culture medium did not differ significantly between control and MJ-treated plants, reaching maximum values at 21 days of treatment (15.87 ± 4.45 and 16.39 ± 2.32 mg of gallic acid equivalents (GAE)/L, respectively). In contrast, CDs and CDs + MJ induced a progressive increase in the TPC of the medium, which at its highest (also at day 21) was 11.5- and 16.8-fold greater compared to the control (184.04 ± -37.55 and 267.68 ± 51.30 mg GAE/L, respectively, Figure 3).

An enhancing effect of CDs on extracellular phenolic compounds has been reported in other plant in vitro cultures. For example, elicitation with 50 mM CDs increased the culture medium content of vanillin, *trans*-resveratrol and total phenolic compounds in *Daucus carota*, *V. vinifera* and *Bryophyllum houghtonii* cultures, respectively [23,42,43]. Treatment with elicitors increases intracellular production of secondary metabolites by triggering a defense response in the plant cell, whereas extractive sequestration by CDs promotes metabolite accumulation in the culture medium. The significant increase in extracellular TPC observed in the present study in CD-treated cultures was likely due to the ability of CDs to host highly hydrophobic molecules in the culture medium.

### 2.3. Stilbene Accumulation in the Culture Medium of Elicited M. alba In Vitro Plants

As shown in Table 1, the major stilbenes identified by HPLC in the culture medium of *M. alba* in vitro plants elicited with 50 mM CDs and/or 100 µM MJ were oxy- and *trans*-resveratrol (Table 1). Levels of oxyresveratrol differed significantly (*p* < 0.001) according to the time and type of elicitation, reaching a maximum when the plants were elicited with CDs alone (5621.95 ± 502.48 µg/L) or in combination with MJ (7738.85 ± 1624.53 µg/L) at day 21, with no significant differences between the two treatments (Table 1). These levels were 11.3-fold higher compared to the treatment with MJ alone (682.70 ± 13.98 µg/L), which suggests that the release of oxyresveratrol into the culture medium was favored by the formation of inclusion complexes with CDs. In contrast, *trans*-resveratrol was only detected in the culture medium supplemented with 50 mM CDs, alone or in combination with 100 µM MJ (109.30 ± 15.97 µg/L and 246.39 ± 18.26 µg/L, respectively), with the levels increasing throughout the experiment until day 21 of elicitation. Therefore, CDs also promoted the release of *trans*-resveratrol into the extracellular medium, although the amounts obtained were far lower compared to oxyresveratrol (Table 1).

### 2.4. Effect of Different Elicitor Concentrations on the Growth of M. alba In Vitro Plants

**Figure 4:**
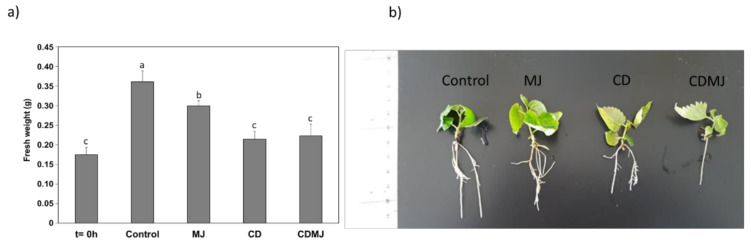
Effect of 5 mM of cyclodextrins (CDs) and/or 10 µM of methyl jasmonate (MJ) on the fresh weight (FW) (**a**) and growth (**b**) of *M. alba* in vitro plants. T=0 h: weight of in vitro plants before elicitor treatment. Different letters denote significant differences between treatments (*p* < 0.05 by post hoc Tukey’s test).

In view of the phytotoxic effect of the treatments (50 mM CDs and/or 100 µM MJ) on the growth of the *M. alba* cultures (Figure 2), a new elicitation experiment was designed with lower concentrations to avoid compromising the survival of the in vitro plants. Thus, the plant cultures were elicited with CDs (5, 12.5 and 25 mM) and MJ (10, 25 and 50 µM) for 7 days. Only the lowest amounts of CDs (5 mM) and MJ (10 µM) had no apparent phytotoxic effects, which increased with concentration (data not shown). For this reason, all experiments were carried out in *M. alba* in vitro plants elicited with 5 mM CDs and 10 µM MJ, separately or in combination. After 7 days of treatment a significant increase in growth with respect to 0 h was only observed in the control and MJ-treated plants, whose fresh weight (FW) increased by 106 and 71%, respectively (Figure 4a). Plants treated with CDs or CDs + MJ showed no significant differences at 7 days compared to 0 h (Figure 4a), except for a lower development of the root system that was more pronounced with the combined treatment (Figure 4b). Therefore, although the lower elicitor concentrations did not have a cytotoxic effect on *M. alba* in vitro plants, growth was inhibited after 7 days of elicitation compared to the control, especially in the presence of CDs.

### 2.5. Effect of 5 mM CDs and/or 10 µM MJ on Total Phenolic Content in Elicited M. alba In Vitro Plants

Compared to the control, the TPC was significantly higher both in the culture medium (Table 2a) and leaves (Table 2b) of *M. alba* in vitro plants treated with CDs or CDs + MJ. Thus, an approximately 5-fold increase in TPC was observed in the culture medium after elicitation with 5 mM CDs or 5 mM CDs + 10 µM MJ (33.483 ± 4.49 and 34.128 ± 2.26 mg GAE/L, respectively) compared to the control (5.65 ± 0.72 mg GAE/L). Likewise, a higher TPC was found in CD-treated leaves (2112.79 ± 174.22 µg EAG/ g FW) than in the control (259.91 ± 16.18 µg GAE/ g FW). The 10 µM MJ treatment increased the TPC in the culture medium, but always to a lesser extent than 5 mM CDs (Table 2a). Furthermore, no significant differences in the TPC of leaves and roots were detected between the 10 µM MJ and control treatments (Table 2b). Once again, the results indicate that the increase in TPC in the culture medium was due to sequestering and secretion activity of CDs, which favored extracellular metabolite accumulation. In contrast, the highest TPC in roots was obtained with the control and MJ treatments (1148.63 ± 228.83 and 1325.79 ± 249.13 µg GAE/ g FW, respectively), with no significant differences between them.

The antioxidant capacity (expressed as Trolox equivalents) in *M. alba* in vitro plants treated with 5 mM CDs and/or 10 µM MJ for 7 days was also measured. As shown in Table 2, the treatments that resulted in the highest TPC in the culture medium and leaves (CDs or CDs + MJ) were also associated with the highest antioxidant capacity (Table 2a,b). In roots, the antioxidant activity was also correlated with the highest TPC, obtained with the control and MJ treatments (Table 2b).

In agreement with these results, CDs have been reported to increase the extracellular antioxidant activity in *Bryophyllum* cell cultures during elicitation, until at day 9 it was 16-fold higher compared to the control treatment [43]. Moreover, the changes were correlated with an increase in the TPC. In a study with *V. vinifera* cell cultures, Almagro et al. [44] found that elicitation with CDs + MJ increased both the intra- and extracellular TPC, and the latter followed the same pattern as the antioxidant activity values. The molecular structure of CDs allows the formation of inclusion complexes with a wide range of molecules, including phenolic compounds [23,43,44]. The complexation of phenolic compounds with CDs not only improves their stability in the culture medium, but also protects them from degradation, which explains the higher extracellular antioxidant activity values found in CD-elicited *M. alba* in vitro plants.

At the end of the 7-day elicitation experiment, extracellular oxyresveratrol and *trans*-resveratrol were only detected in the CD-treated plants (Table 2a), whose leaves also contained both stilbenes but at lower levels (Table 2b); neither stilbene was found in roots (Table 2b). Notably, in both leaves and culture medium, the accumulation of oxyresveratrol was higher compared to its precursor, *trans*-resveratrol, which undergoes hydroxylation to produce oxyresveratrol. Komaikul et al. [34] reported that treatment with 2-hydroxypropyl-β-CDs increased the accumulation of oxyresveratrol and resveratrol in the culture medium of free *M. alba* callus compared to the control (730- and 43-fold, respectively). In an elicitation experiment with *M. alba* root cultures [32], the highest levels of oxyresveratrol (68.6 ± 3.53 μg/g dry weight (DW)) and resveratrol (10.2 ± 0.53 μg/g DW) were obtained using 200 μM MJ and 2 mg/mL yeast extract. In a previous study with *M. alba* immobilized cells [33], elicitation with 50 μM MJ and 0.5 mg/mL yeast extract for 72 h also triggered an increase in resveratrol and oxyresveratrol, which reached levels of 140 and 65 μg/g DW, respectively. In all cases, the production of oxyresveratrol was higher than that of resveratrol, as in the present study. Therefore, the elicitation of *M. alba* in in vitro cultures activates the phenylpropanoid pathway leading to the biosynthesis of stilbenes such as oxyresveratrol and resveratrol. Furthermore, the presence of CDs promotes not only the production of these metabolites but also their accumulation in the extracellular medium.

## 3. Materials and Methods

### 3.1. Plant Materials

*Morus alba* cv. Cristiana was kindly provided by Dr. José Luis Cenis from the Instituto Murciano de Investigaciones Agrarias y Alimentarias (IMIDA), Spain. Under aseptic conditions, young branches containing at least 5 axillary buds were immersed in 70% ethanol for 1 min and surface-disinfected with 20% sodium hypochloride solution containing 0.1% Tween 20 for 15 min. After removing the disinfectant agent, vegetative nodal segments were deposited on Murashige and Skoog (MS) basal medium [45] supplemented with 250 mg/L of casein hydrolysate, 30 g/L of sucrose, Morel’s vitamins [46], and 8 g/L agar at 6 pH. The glass tubes containing the nodal segments were kept at 25 °C under a 16 h light/8 h dark photoperiod with a photon flux density of 85 µmol m^2^/s, and a relative humidity of 60 ± 2%. In vitro plants were maintained by vegetative multiplication in the agar-solidified medium described above.

### 3.2. Elicitation Treatments of M. alba In Vitro Plants

Joint elicitation with 100 µM MJ and 50 mM CDs has been described as an effective strategy to increase secondary metabolite production in various plant cell cultures [23,24,26,47,48]. Thus, to evaluate the effect of this treatment on stilbene production in *M. alba*, the plants were treated with 50 mM CDs and/or 100 µM MJ, using the culture medium described above without agar. CDs were added to the culture medium before autoclaving, whereas MJ was sterilized by filtration, dissolved in ethanol, and added to the medium after autoclaving. Nodal segments of in vitro plants, which contained at least 2 leaves, were elicited for 7, 14 or 21 days to determine the optimal elicitation time. After each elicitation period, the culture medium was separated from the in vitro plants and the following parameters were determined: volume of spent medium and fresh weight (FW). All experiments were performed in quadruplicate.

#### Optimization of Stilbene Production in *M. alba* In Vitro Plants Treated with Cyclodextrins and Methyl Jasmonate

To optimize stilbene production, *M. alba* in vitro plants were elicited with 5 mM CD and/or 10 µM MJ, and maintained at the aforementioned temperature, photoperiod and humidity for 7 days, after which stilbenes were extracted from the spent medium, leaves and roots. All experiments were performed in quadruplicate.

### 3.3. Extraction of Stilbenes

Stilbenes were extracted from the elicited culture medium by phase partitioning with ethyl acetate (1:1, *v/v*) as described by Sabater-Jara and Pedreño [27]. To extract the metabolites from plant material (leaves and roots), 100 mg (FW) was homogenized with 80% methanol (1:4, *w/v*) for 30 min at 80 °C and the samples were centrifuged at 13.000× *g* for 5 min. This process was repeated twice. The methanol was then evaporated in vacuum as described by Sabater-Jara and Pedreño [27]. The dry extract was dissolved in 1 mL methanol for chromatographic analysis.

### 3.4. Identification and Quantification of Stilbenes

#### 3.4.1. Total Phenolic Content

The soluble TPC was determined by the Folin–Ciocalteu method [49]. For quantitative analysis an external standard calibration curve for gallic acid (assay ≥ 99%; Sigma-Aldrich, Hamberg, Germany), ranging from 0.01 to 10 µg/mL, was used. The calibration curve was y = 0.0819x + 0.0004; R² = 0.999. The results were expressed as GAE per L for the elicited culture medium or g FW for M. alba leaves and roots.

#### 3.4.2. HPLC-DAD Analysis of Stilbenes

The individual stilbene compounds were analyzed using high-performance liquid chromatography with diode-array detection (HPLC-DAD) (Jasco LC-Netll/ADC) on a Zorbax SB-C18 column at room temperature. The mobile phase consisted of solvent A (acetic acid pH 2.5) and solvent B (acetonitrile), with the following gradient: 0 min, 85% solvent A; 5 min, 80% solvent A; 10–15 min, 65% solvent A; 17–25 min, 10% solvent A; and 25–30 min, 65% solvent A; 30–35 min, 85% solvent A. The flow rate was 1 mL/min. Stilbene identification and quantification was performed using external standard calibration curves for oxyresveratrol (HPLC ≥ 97%; Sigma-Aldrich, Hamberg, Germany) and *trans*-resveratrol (HPLC ≥ 99%; Sigma-Aldrich, Hamberg, Germany). The standard calibration curves for oxyresveratrol (y = 32425x + 13673; R² = 0.999) and *trans*-resveratrol (y = 80240x + 123593; R² = 0.997) were prepared using a concentration ranging from 10 to 500 µg/mL and 1 to 50 µg/mL for oxy- and *trans*-resveratrol, respectively. All experiments were performed in triplicate.

### 3.5. Antioxidant Activity

The antioxidant properties of stilbene-enriched extracts from in vitro *M. alba* plants were determined using the Trolox equivalent antioxidant capacity (TEAC) method or ABTS method, in which the effect of stilbene-enriched extracts on the stable free radical ABTS^·+^ [2,2′-azino-bis (3-ethylbenzothiazoline-6-sulfonic acid)] was analyzed as described by Gandía-Herrero et al. [50,51]. In the TEAC method, Trolox (assay ≥ 99%; Sigma-Aldrich, Hamberg, Germany) was used as the external standard and the calibration curve was y = 0.0413x + 0.0584; R² = 0.9819, ranging from 0 to 12 µM. The antioxidant capacity was expressed as mM Trolox per L for the elicited culture medium or g FW for leaves and roots.

### 3.6. Statistical Analysis

An analysis of variance (ANOVA) was tested by Tukey’s honestly significant difference test using the Statistical Package for the Social Sciences software version 22 (SPSS Inc., Chicago, IL, USA). Differences were considered statistically significant at *p* < 0.05.

## 4. Conclusions

In this study of bioactive compound production in *M. alba* in vitro plants, elicitation with 5 mM CDs alone or in combination with 10 µM MJ resulted in a significant increase in TPC in both the culture medium and leaves after 7 days of treatment, without compromising plant viability. The TPC of roots was higher in the control and MJ-treated plants, suggesting that CDs promote the release of phenolic compounds biosynthesized in the roots. By a mechanism of extractive sequestration, CDs allow these highly hydrophobic metabolites to accumulate in aqueous culture media. Moreover, the increase in TPC in *M. alba* in vitro plants treated with CDs with or without MJ was correlated with an increase in the antioxidant capacity of the extracts. The bioactive stilbenes oxy- and *trans*-resveratrol were found in the culture medium and leaves of *M. alba* in vitro plants elicited with CDs or CDs + MJ, but not in the roots. The highest levels of these stilbenes were correlated with the highest TPC, and antioxidant capacity detected in CD-treated plants. Thus, elicited *M. alba* in vitro plants constitute not only an environmentally friendly and sustainable source of valuable bioactive compounds for biotechnological production, but also the use of this new protocol could be of interest for studying plant defense mechanisms in bioprotection.

## Figures and Tables

**Table 1:** Levels of oxy- and *trans*-resveratrol in the culture medium of *M. alba* in vitro plants elicited with 50 mM cyclodextrins (CDs) and/or 100 µM methyl jasmonate (MJ) after 7, 14 and 21 days of treatment. Different letters indicate a significant difference between the means in each treatment (*p* < 0.05 by post hoc Tukey’s test). nd: not detected.

Elicitation time (d)	Treatment	oxyresveratrol (µg/L)	*trans*-resveratrol (µg/L)
7	Control	nd	nd
	MJ	162.63 ± 37.71 ^b^	nd
	CD	948.56 ± 146.81 ^b^	nd
	CDMJ	4180.48 ± 1552.17 ^a^	62.24 ± 18.99
14	Control	103.27 ± 9.98 ^c^	nd
	MJ	323.52 ± 139.80 ^c^	nd
	CD	3301.93 ± 392.78 ^b^	40.03 ± 8.22 ^a^
	CDMJ	9542.87 ± 230.82 ^a^	47.69 ± 3.16 ^a^
21	Control	nd	nd
	MJ	682.70 ± 13.98 ^b^	nd
	CD	5621.95 ± 502.48 ^a^	109.30 ± 15.97 ^b^
	CDMJ	7738.85 ± 1624.53 ^a^	246.39 ± 13.26 ^a^

**Table 2:** Total phenolic (TPC) and oxy- and *trans*-resveratrol content and antioxidant capacity in the culture medium (a) and leaves and roots (b) of *M. alba* in vitro plants elicited with 5 mM CD, 10 µM MJ or both (CDMJ) for 7 days. Different letters denote a significant difference between the means in each treatment (*p <* 0.05 by post hoc Tukey’s test). nd: not detected.

(a)	Treatment	mgGAE/L	µg oxyresveratrol/L	mM Trolox/L
Culture media	Control	5.65 ± 0.72 ^c^	nd	65.84 ± 4.10 ^d^
	MJ	10.62 ± 1.48 ^b^	nd	160.27 ± 20.57 ^c^
	CD	34.13 ± 2.26 ^a^	231.66 ± 78.63 ^b^	684.49 ± 55.39 ^a^
	CDMJ	33.48 ± 4.49 ^a^	316.98 ± 13.83 ^a^	486.70 ± 4.45 ^b^
**(b)**	**Treatment**	**µgGAE/gFW**	**µg oxyresveratrol/g FW**	**mM Trolox/g FW**
Leaves	Control	259.91± 16.18 ^b^	nd	10.07 ± 2.36 ^c^
	MJ	362.69 ± 31.41 ^b^	nd	4.91 ± 0.59 ^d^
	CD	2112.79 ± 174.22 ^a^	16.90 ± 3.43 ^a^	23.26 ± 1.89 ^b^
	CDMJ	1818.20 ± 414.94^a^	20.70 ± 6.29 ^a^	28.70 ± 2.29 ^a^
Roots	Control	1148.83 ± 228.83 ^a^	nd	35.76 ± 3.90 ^a^
	MJ	1325.79 ± 249.13 ^a^	nd	30.26 ± 2.23 ^a.b^
	CD	738.40 ± 232.09 ^b^	nd	1.86 ± 0.13 ^c^
	CDMJ	122.10 ± 22.76 ^c^	nd	25.70 ± 9.82 ^b^

## Data Availability

The data are contained within the article.
